# The Nordic Aortic Valve Intervention (NOTION) trial comparing transcatheter versus surgical valve implantation: study protocol for a randomised controlled trial

**DOI:** 10.1186/1745-6215-14-11

**Published:** 2013-01-09

**Authors:** Hans Gustav Thyregod, Lars Søndergaard, Nikolaj Ihlemann, Olaf Franzen, Lars Willy Andersen, Peter Bo Hansen, Peter Skov Olsen, Henrik Nissen, Per Winkel, Christian Gluud, Daniel Andreas Steinbrüchel

**Affiliations:** 1 Department of Cardiothoracic Surgery, The Heart Centre, Rigshospitalet, Copenhagen University Hospital, Blegdamsvej 9, DK 2100, Copenhagen, Denmark; 2 Department of Cardiology, The Heart Centre, Rigshospitalet, Copenhagen University Hospital, Blegdamsvej 9, DK 2100, Copenhagen, Denmark; 3 Department of Cardiac Anesthesia, The Heart Centre, Rigshospitalet, Copenhagen University Hospital, Blegdamsvej 9, DK 2100, Copenhagen, Denmark; 4 Department of Cardiology, Odense University Hospital, Sdr. Boulevard 29, DK 5000, Odense, Denmark; 5 Copenhagen Trial Unit, Centre for Clinical Intervention Research, Rigshospitalet, Copenhagen University Hospital, Tagensvej 22, DK 2100, Copenhagen, Denmark

**Keywords:** Aortic valve stenosis, Aortic valve prosthesis, Transcatheter aortic valve implantation, Surgical aortic valve replacement, Randomised clinical trial design

## Abstract

**Background:**

Degenerative aortic valve (AV) stenosis is the most prevalent heart valve disease in the western world. Surgical aortic valve replacement (SAVR) has until recently been the standard of treatment for patients with severe AV stenosis. Whether transcatheter aortic valve implantation (TAVI) can be offered with improved safety and similar effectiveness in a population including low-risk patients has yet to be examined in a randomised setting.

**Methods/Design:**

This randomised clinical trial will evaluate the benefits and risks of TAVI using the transarterial CoreValve System (Medtronic Inc., Minneapolis, MN, USA) (intervention group) compared with SAVR (control group) in patients with severe degenerative AV stenosis. Randomisation ratio is 1:1, enrolling a total of 280 patients aged 70 years or older without significant coronary artery disease and with a low, moderate, or high surgical risk profile. Trial outcomes include a primary composite outcome of myocardial infarction, stroke, or all-cause mortality within the first year after intervention (expected rates 5% for TAVI, 15% for SAVR). Exploratory safety outcomes include procedure complications, valve re-intervention, and cardiovascular death, as well as cardiac, cerebral, pulmonary, renal, and vascular complications. Exploratory efficacy outcomes include New York Heart Association functional status, quality of life, and valve prosthesis and cardiac performance. Enrolment began in December 2009, and 269 patients have been enrolled up to December 2012.

**Discussion:**

The trial is designed to evaluate the performance of TAVI in comparison with SAVR. The trial results may influence the choice of treatment modality for patients with severe degenerative AV stenosis.

**Trial registration:**

ClinicalTrials.gov: NCT01057173

## Background

Degenerative aortic valve (AV) stenosis is the most prevalent heart valve disease in the western world, affecting 2 to 7% of people older than 65 years of age. Both the incidence and prevalence are expected to rise due to the general increase in life expectancy [1,2]. The disease has a chronic course and carries a poor prognosis after onset of symptoms [3]. Medical therapy offers sparse symptomatic relief, and symptomatic patients have a 2-year survival of approximately 50%. Surgical aortic valve replacement (SAVR) is an effective treatment, and complication rates and prosthesis durability are well known. The surgical procedure carries a low operative risk in younger patients without any significant co-morbidity (30-day mortality 3 to 5% for age <70 years). This risk increases substantially with increasing age, reduced left ventricular function, and other co-morbidities (30-day mortality 5 to 15%) [4,5]. Presumably because of this increase, almost one-third of patients referred for valve intervention at the time of trial design did not receive valve replacement and were continued on medical therapy [6]. A less invasive treatment option would therefore be attractive.

Different operative risk calculators in cardiac surgery (the Society of Thoracic Surgery Predicted Risk of Mortality (STS) score [7] and the European System for Cardiac Operative Risk Evaluation (EuroSCORE) [8]) have been developed to identify the high-risk surgical patient, but these are inadequate and generally overestimate the operative mortality in SAVR. A new EuroSCORE II has been developed and is being considered for implementation [9]. Data from randomised clinical trials are needed to improve treatment decision-making.

In recent years a new treatment option has become available for patients considered at high risk for SAVR. Transcatheter aortic valve implantation (TAVI) was originally developed in 1992 by Andersen and coworkers and was clinically introduced in 2002 by Cribier and coworkers as a minimally invasive treatment for patients considered ineligible for valve surgery [10,11]. The term TAVI comprises different valve prosthesis types, techniques, and approaches to the stenosed valve. Originally developed as a transvenous transseptal septal technique, the procedure is currently performed on the beating heart either antegrade transapically through a small left anterior thoracotomy or retrograde through the arterial system using either the femoral, subclavian, or carotid artery, or with the direct transaortic approach [12,13]. The artery can be surgically exposed or punctured. The two most widely used TAVI systems, both Conformité Européenne (European Conformity) marked, are the Edwards SAPIEN with a balloon-expandable bioprosthesis (Edwards Lifescience Inc., Irvine, CA, USA) and the CoreValve System with a self-expandable bioprosthesis (Medtronic Inc., Minneapolis, MN, USA). The former has also been Food and Drug Administration approved in the United States.

Postoperative complications related to SAVR stem from sternotomy, cardiopulmonary bypass, aortic cross-clamping, and cardioplegic cardiac arrest. In TAVI many of the SAVR complications are potentially avoided, but others are potentially exacerbated such as neurological lesions (2 to 11% during the first year) [14,15]. Retrograde catheter passage in the aortic arch and ascending aorta, and native valve pre-dilatation – causing calcified and often ulcerated leaflets to fracture, exposing thrombogenic endothelial lesions to the circulation – could generate atherosclerotic emboli. At the same time, TAVI-specific complications have been encountered – including haemodynamic instability and arrhythmia during implantation, prosthesis misplacement, and incomplete frame expansion (leading to prosthesis embolisation, valvular and paravalvular leakage, and valve-in-valve implantation, or conversion to open surgery), aortic and ventricular perforations, and arterial access lesions [16]. Furthermore, the CoreValve System has a high frequency of conduction abnormalities (atrioventricular and left bundle branch block) requiring a permanent pacemaker in 20 to 30% of patients, presumably because of septal compression [17]. Since operator experience has grown and implantation systems have improved and become smaller, many of these complications have become less frequent.

National and international registries have documented good short-term and mid-term safety results after TAVI in patients considered at high risk for surgery with a 95% procedure success rate, and with 30-day mortality, stroke, and myocardial infarction rates of 5 to 12%, 2 to 10%, and 1 to 4%, respectively, combined with excellent sustained prosthesis function, and clinical improvements [15,16,18-20]. Long-term results are lacking, but no reports of fractured prosthetic stents or frames have been published. Anecdotal reports of calcified TAVI prostheses after 5 years have been published [21].

Results after 1 and 2 years from the randomised trial (PARTNER Trial) comparing the Edwards SAPIEN prosthesis with standard medical therapy in patients considered to have prohibitive high surgical risk (Cohort B), and with SAVR in patients with high surgical risk (Cohort A) have been published [22-25]. For Cohort B an all-cause mortality reduction within the first year of 20% was observed (TAVI vs. medical therapy, 30.7% vs. 50.7%; *P* <0.001), and for Cohort A non-inferiority for all-cause death after 1 year was proven (TAVI vs. SAVR, 24.2% vs. 26.8%; *P* = 0.44). Complication rates were significantly different, with more strokes and vascular injuries in the TAVI group but more major bleedings and more new-onset atrial fibrillation in the SAVR group. Another randomised trial using the Edwards SAPIEN system including low-risk patients has been reported. This trial was prematurely terminated after including 70 patients because of a nonsignificant excess of events in the TAVI group [26].

No randomised trials comparing TAVI versus SAVR in patients with low, moderate, and high operative risk and using the CoreValve System have been reported. Typically, high-risk patients considered for TAVI in current clinical practice have a logistic EuroSCORE >20% and STS score >10% predicted 30-day operative mortality. Low-risk patients could have a logistic EuroSCORE of 3% and a STS score of 1%. Patient preferences, the less invasive technique, and unclear indications could promote off-label use of TAVI, as has been seen in percutaneous coronary intervention. A randomised clinical trial can examine this new treatment option more extensively and generate evidence needed to broaden, optimise, and guide treatment for patients with severe degenerative AV stenosis [27].

## Methods/Design

### Trial design and objectives

The Nordic Aortic Valve Intervention (NOTION) trial is a multicentre, randomised clinical trial without blinding comparing TAVI using the CoreValve System versus SAVR in the treatment of severe AV stenosis designed during 2008/09 and launched in December 2009. The primary objective is to evaluate the safety and effectiveness of TAVI using the CoreValve System compared with SAVR using cardiopulmonary bypass.

The regional ethics committee has approved the trial at each clinical site, and all patients will provide written informed consent. The trial is conducted according to the Declaration of Helsinki. The organisational and coordinating site is The Heart Centre, Rigshospitalet, Copenhagen University Hospital, Copenhagen, Denmark, and the regional Research Ethics Committee in The Capital Region of Denmark approved the protocol. Other clinical sites are Odense University Hospital, Odense, Denmark, and Sahlgrenska University Hospital, Gothenburg, Sweden. The trial is registered at ClinicalTrials.gov (NCT01057173).

The trial is investigator initiated, designed, and conducted with no sponsor involvement. The trial has a steering committee, an independent event adjudication committee, and an external independent data monitoring and safety committee (DMSC). The DMSC is composed of an interventional cardiologist, a cardiac surgeon, a neurologist, and a statistician.

### Device and procedures

**Figure 1:**
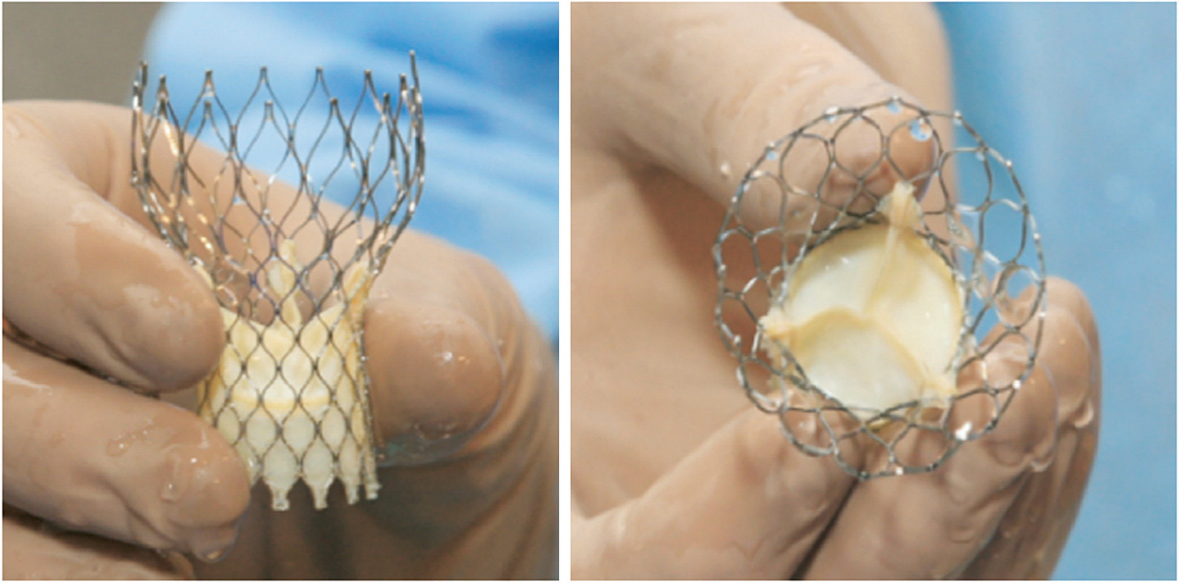
**The transcatheter aortic valve implantation bioprosthesis.** The third-generation porcine pericardial trileaflet valve mounted in a self-expanding nitinol support frame (CoreValve System; Medtronic Inc., Minneapolis, MN, USA). TAVI, transcatheter aortic valve implantation.

The CoreValve System is a TAVI system designed to treat AV stenosis [20]. The procedure can be done on the beating heart with a retrograde arterial access. The third-generation system used for this trial includes a valve bioprosthesis consisting of a porcine pericardial trileaflet valve mounted in a nitinol self-expandable frame (frame diameter size 23, 26, 29, or 31 mm covering an aortic annulus diameter from 18 to 29 mm, length 52 to 55 mm; Figure 1), and an 18 French (6 mm) delivery system that enables implantation of the prosthesis. The TAVI procedure is performed under local or general anaesthesia in the cardiac catheterisation laboratory, either percutaneously via the femoral artery or after surgical cut-down of the subclavian artery. Before implantation, the native AV is pre-dilated with a balloon valvulotomy during rapid ventricular pacing to ensure a stable position of the balloon. The prosthesis is crimped in the delivery catheter. During deployment, the prosthetic frame will expand to its preformed shape and lodge itself in the left ventricular outflow tract, the aortic annulus, and the ascending aorta. The prosthesis can be retrieved until fully deployed, and an intervention catheter can pass through the frame to the coronary ostia. The access vessel is closed either with a percutaneous suture system (ProStar XL or Perclose ProGlide; Abbott Vascular, Inc., Menlo Park, CA, USA) or surgically. Before implantation a temporary pacemaker lead is placed in the right ventricle, used for rapid pacing and in case of conduction abnormalities. Correct prosthesis placement during and after deployment is verified by fluoroscopy and echocardiography. Before the procedure, patients receive a loading dose of 300 mg clopidogrel. During the procedure, intravenous antibiotics are given before skin puncture, and 5,000 units heparin are given after vascular access has been established. After the procedure, patients receive 75 mg clopidogrel daily for 3 months as well as 75 mg acetyl salicylic acid lifelong. If vitamin K antagonist therapy is indicated, this is added to the above treatment. Other cardiac medications are prescribed according to local cardiologic guidelines.

SAVR is performed using standard surgical techniques and general anaesthesia. The procedure includes a complete median sternotomy, cardiopulmonary bypass in normothermia, aortic cross-clamping, and cold blood cardioplegic cardiac arrest. The native valve is excised and the annulus is carefully decalcified and irrigated before a bioprosthesis is sutured in place. The choice of type and size of the prosthesis is at the discretion of the surgeon. Radiofrequency ablation will be performed for chronic or intermittent atrial fibrillation. Postoperative antiplatelet and other cardiac medical therapy is similar to the TAVI group.

After the procedure, both patient groups will be admitted to a specialised cardiac intensive care unit at least overnight, and TAVI patients will thereafter be transferred to the cardiology ward and SAVR patients to the cardiac surgery ward.

### Patient population

Eligible patients include those aged 70 years or older with severe degenerative AV stenosis with symptoms or without symptoms but with left ventricular systolic dysfunction and/or hypertrophy (see Table 1 for all inclusion and exclusion criteria). Patients must be suitable for both TAVI and SAVR according to a cardiac surgeon, an interventionist, and an echocardiographer at a multidisciplinary conference. Inclusion criteria follow general surgical indications and the TAVI system manufacturer’s guidelines for severe AV stenosis. Patients with previous heart surgery, other significant valve disease, or coronary artery disease requiring revascularisation at the time of referral are excluded. Patients with a stroke or transient ischemic attack within the previous 30 days or an acute coronary syndrome within the previous year are also excluded.

**Table 1:** Inclusion and exclusion criteria

**Inclusion criteria**	
•	Severe degenerative AV stenosis (calcified AV, effective orifice area <1 cm^2^ or indexed for body surface area <0.6 cm^2^/m^2^, mean AV gradient >40 mmHg, or AV peak systolic velocity >4.0 m/second), and
•	Symptomatic (dyspnoea ≥NYHA class II, angina pectoris, or syncope), or
•	Asymptomatic with one or more of the following:
	• Left ventricle posterior wall thickness ≥17 mm
	• Left ventricular ejection fraction <60% but ≥20%
	• Atrial fibrillation
•	Age ≥70 years
•	Candidate (clinical and anatomical) for both TAVI and SAVR (as specified by prosthesis manufacture’s guidelines) judged by a multidisciplinary conference
•	Expected to survive ≥1 year after intervention
•	Able to provide written informed consent
**Exclusion criteria**	
•	Isolated AV insufficiency
•	Other significant cardiac valve or septal diseases
•	Coronary artery comorbidity requiring revascularisation (PCI or CABG)
•	Intracardiac lesion (thrombus, tumour, vegetation)
•	Previous open cardiac surgery
•	Myocardial infarction or PCI within the last year
•	Stroke or transient ischemic attack within the last 30 days
•	Renal insufficiency requiring haemodialysis
•	Pulmonary insufficiency (FEV1 or diffusion capacity <40% of expected)
•	Active infectious disease requiring antibiotics
•	Emergency intervention (within 24 hours after the indication for intervention has been made)
•	Unstable pre-interventional condition requiring inotropic support or mechanical cardiac assistance
•	A known hypersensitivity or contraindication to nitinol, heparin, clopidogrel, acetyl salicylic acid, or contrast material
•	Currently participating in an investigational drug or another device study

AV, aortic valve; CABG, coronary artery bypass grafting; FEV1, forced expiratory volume in 1 second; NYHA, New York Heart Association; PCI, percutaneous coronary intervention; SAVR, surgical aortic valve replacement; TAVI, transcatheter aortic valve implantation.

### Patient screening, randomisation, and follow-up

**Figure 2:**
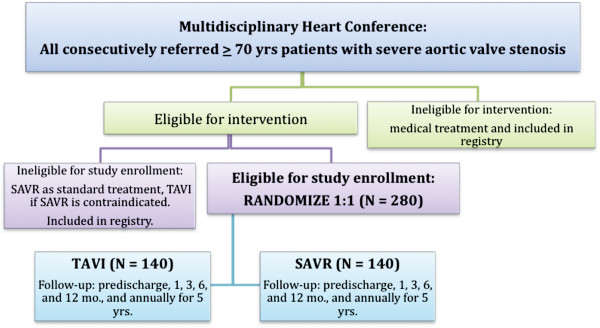
**Study flow chart.** An allcomers trial design. SAVR, surgical aortic valve replacement; TAVI, transcatheter aortic valve implantation.

All patients consecutively referred for SAVR at each study centre (all tertiary university hospitals) have been screened since December 2009 for eligibility at a multidisciplinary conference (Figure 2). Patients determined as ineligible are recorded for registries and follow-up. Potential eligible patients are interviewed and informed of trial risks and objectives before obtaining informed consent. Transthoracic echocardiography (transoesophageal studies are done if appropriate), coronary, aortic, and femoro-iliac angiograms, lung function tests, and standard laboratory blood samples are obtained in order to determine eligibility. Electrocardiogram (ECG)-gated high-resolution computed tomography studies can be used to evaluate the aortic annulus and root, and non-ECG-gated studies can further evaluate the arterial system.

The trial will be conducted at three sites in Scandinavia and will enrol up to 280 patients. Randomisation will be 1:1 with approximately 140 patients in each group (TAVI and SAVR) and stratified according to age (70 to 74 years or ≥75 years), coronary status (coronary artery disease not requiring revascularisation or normal), and trial site. Randomisation is central at the Copenhagen Trial Unit, which has generated the allocation sequence in permuted blocks with unknown block size for the investigators.

Patients undergo the assigned treatment with either TAVI using the CoreValve System or SAVR. Clinical follow-up with outcome measure recording, blood samples, and ECG occurs at pre-discharge and at 1, 3, 6, 12 months, and annually for a minimum of 5 years. Transthoracic echocardiography studies are performed before discharge and after 3 and 12 months, and annually. Patients will furthermore be followed in external electronic medical records and retrieved medical records for hospital admissions, the Danish National Hospital Registry [28], the Civil Registration System [29], and the Registry of Causes of Death [30].

### Outcomes

The primary outcome is the composite of myocardial infarction, stroke, or all-cause mortality within the first 12 months (Table 2). Formal neurological evaluations and computed tomography cerebral studies will only be performed when clinically indicated and not routinely. ECGs and coronary markers will be evaluated routinely the first 3 days after the intervention and when indicated.

**Table 2:** Outcomes, variable type, time/period of measurement, and statistical analysis for primary and exploratory outcomes

**Outcome**^**a**^	**Type of variable**	**Time or period of measurement**	**Statistical type of analysis**^**b**^
Myocardial infarction, stroke, or all-cause mortality (P)	Binary	During 12 months	1
Myocardial infarction (E)	Binary	During 30 days. During 12 months	1
Stroke (E)	Binary	During 30 days. During 12 months	1
All-cause mortality (E)	Binary	During 30 days. During 12 months	1
Cardiovascular mortality (E)	Binary	During 30 days. During 12 months	1
Prosthesis re-intervention (E)	Binary	During 30 days. During 12 months	1
Conduction abnormality requiring pacemaker (E)	Binary	During 30 days. During 12 months	1
Arrhythmia (E)	Binary	During 30 days. During 12 months	1
Endocarditis (E)	Binary	During 30 days. During 12 months	1
Mechanical ventilation in ≥24 hours (E)	Binary	During 30 days	1
Acute kidney injury (stage 2 or 3) (E)	Binary	During 30 days	1
Access-site injury (major) (E)	Binary	During 30 days	1
Bleeding (major–minor) (E)	Ordered	During 30 days	3
Number of days hospitalised (E)	Discrete	During 12 months	2
NYHA functional class (I to IV) (E)	Ordered^c^	At 3 months and at 12 months	3
SF-36 Quality of life (E)	Continuous^c^	At 3 months and at 12 months	4
Effective orifice area (E)	Continuous^c^	At 3 months and at 12 months	4
Prosthesis peak gradient (E)	Continuous^c^	At 3 months and at 12 months	4
Prosthesis mean gradient (E)	Continuous^c^	At 3 months and at 12 months	4
Prosthesis regurgitation (E)	Ordered^d^	At 3 months and at 12 months	3
Prosthesis–patient mismatch (severe) (E)	Binary	At 3 months and at 12 months	1
Left ventricular ejection fraction (E)	Continuous^c^	At 3 months and at 12 months	4

P, primary outcome; E, exploratory outcome; NYHA, New York Heart Association; SF-36, Short-form health survey with 36 questions. ^a^Outcomes defined according to Valve Academic Research Consortium criteria. ^b^See analytical procedures under Statistical analysis plan. ^c^ Baseline value is measured and is included in the adjusted analysis. ^d^Graded as none, mild, moderate, or severe.

Exploratory safety outcomes at 30 days and 12 months include all-cause mortality; cardiovascular mortality; cardiac and prosthetic AV complications (myocardial infarction; new-onset arrhythmias and conduction abnormalities; prosthetic AV re-intervention, and endocarditis); stroke (ischaemic and haemorrhagic). Exploratory safety outcomes at 30 days include pulmonary, renal, access-related vascular and bleeding complications. Exploratory safety outcomes at 12 months include number of days hospitalised.

Exploratory patient-oriented and prosthetic AV performance outcomes at 3 and 12 months include New York Heart Association functional class, quality of life (Short-form health survey with 36 questions questionnaire), and prosthetic AV opening area, peak and mean pressure gradients, prosthetic–patient mismatch, and regurgitation, as well as left ventricular function.

All outcomes will be assessed and recorded before discharge and at all follow-up visits.

Definitions of outcomes are adopted from the generally accepted Valve Academic Research Consortium consensus report for TAVI clinical trials [31,32].

Evaluation of the primary outcome measure by the independent event adjudication committee will be blinded to the intervention performed. The blinding consists of removal of all information related to the type of intervention and prosthesis from collected patient records. The evaluation of exploratory outcomes, except for quality of life data, is difficult to blind since procedure and prosthesis types can be identified.

All required trial data and outcomes will be collected on standardised patient report forms and transferred to a central database for storage. The principal investigators at the organising trial site will manage and prepare data for publication, and an external independent statistician will perform the statistical analysis.

### Sample size calculation

The trial is designed as a randomised superiority trial with the alternative hypothesis that TAVI is better than SAVR regarding the effect on the primary composite outcome consisting of myocardial infarction, stroke, or all-cause mortality after 1 year of follow-up. As the primary outcome measure is a binary variable, the sample size may be estimated using a chi-square test with one degree of freedom and an equal number of patients in each treatment group. Based on the estimated occurrence of the primary outcome measure of 15% in the SAVR group and 5% in the TAVI group during the first year, corresponding to an absolute risk reduction of 10% or a relative risk reduction of 66.7%, and a chosen power of 1 – β = 80% and two-sided α = 5%, then 280 patients (140 patients per intervention group) are needed.

### Statistical analysis plan

All analyses will be intention-to-treat analyses performed blinded with two-sided tests and 5% level of significance. Table 2 shows for each outcome its priority (primary or to be used for explorative analyses), the time at which or the period during which it will be measured, the type of variable, and the analytical procedure (see below) to be used.

#### Analytical procedures

Depending on type of outcome measure, one of four types of regression analyses will be applied. The primary analytical results are those adjusted for age, trial site, presence of coronary disease not requiring revascularisation, and baseline value (possibly following multiple imputations; see below). Unadjusted analyses will also be carried out and major discrepancies between the results of adjusted and unadjusted analyses will be discussed. The types of regression analyses are as follows.

Type 1: Logistic regression. In case of lack of convergence fit or non-estimable odds ratios, Fisher’s exact test will be used and the protocol specified covariates thus disregarded.

Type 2: Includes a count of events within a specified period (12 months). Using the countreg procedure (SAS software version 9.3, SAS Institute Inc., Cary, NC, USA), the Poisson model, the negative binomial model, the zero inflated Poisson model, and the zero inflated negative binomial model will be compared by testing for overdispersion and comparing the average predicted count probabilities and the observed proportions. The best model will be used to analyse the count data. As a sensitivity analysis, a nonparametric test (Mann–Whitney test) will be conducted to compare the distributions of counts between the groups and the results will be discussed.

Type 3: Regression analysis using the proportional odds model for ordered variables. If the model assumption is significantly violated, then the Mann–Whitney test will be used.

Type 4: The general linear univariate model will be used. As a sensitivity analysis, a nonparametric test (Mann–Whitney test) will be conducted and the results discussed.

An explorative time-to-event analysis based on all available follow-up data on mortality will be conducted using Kaplan–Meier estimates and comparisons between groups with the use of the log-rank test.

#### Missing values

If the missingness exceeds 5% or the result of Little’s test is significant (*P* <0.05), then multiple imputations will be used (SPSS software version 17 or later, IBM Corp., Armonk, NY, USA). Ten imputed datasets will be generated and used.

The potential for bias caused by values missing not at random will be assessed for the primary outcome as a sensitivity analysis as follows: let A be the group with a beneficial effect of intervention as compared with the other group B. The missing values in A will be imputed by 1 (signifying that an event occurred) and those in group B by 0.

#### Multiplicity

All outcomes apart from the primary outcome measure are exploratory and hypothesis generating.

The DMSC will conduct an interim analysis after the first 20 primary outcomes have occurred, which may prompt the DMSC to advise the steering committee to terminate or modify the trial if a significant difference is likely. The interim analyses are based on a statistical approach defined in the charter for the work of the DMSC. A random sample of 10% of patients will have their data monitored with source data verification by the regional good clinical practice unit. All statistical analysis will be performed with the use of SPSS or SAS software.

## Discussion

The design of the NOTION trial is unique in that no randomised comparisons have been made between TAVI with the CoreValve System versus SAVR in a cohort of low-risk, moderate-risk, and high-risk patients. The trial is designed to determine whether TAVI is safer than SAVR with similar efficacy in reducing AV stenosis. Favourable TAVI registry data are expanding, but randomised data are lacking. Despite this and high treatment costs, the number of TAVI procedures is increasing rapidly.

Designing a trial comparing TAVI versus SAVR is challenging. Complication types and their rates following endovascular and surgical procedures are different and must be weighed quantitatively as well as qualitatively. There was very little experience with TAVI in low-risk and moderate-risk patients and no randomised trials involving TAVI at the time of the design of this trial during 2008/09, so the expected rates of outcome measures are therefore based on clinical judgement, in-hospital complication databases, registry data, STS score, and EuroSCORE. Consequently, there is a degree of uncertainty in estimating the trial sample size as well as calculating power for the exploratory outcomes.

Of particular concern during and after TAVI is the possible elevated risk of stroke, unknown durability of the prosthesis, and the long-term cardiac effect of the frequent paravalvular leakage and conduction abnormalities. High-risk patients have clinical characteristics (for example, previous stroke, peripheral vascular disease, hypertension) that increase the risk of stroke and other complications with any major cardiovascular intervention. Less morbid patients with a good functional pre-intervention status are likely to benefit even more from TAVI than these high-risk patients, and complication rates are expected to decline. However, no safety data exist for this particular patient population. The decision to include this low-risk group of patients in the trial therefore presents a dilemma, since balancing the risk of the abovementioned complications and expanding our knowledge of this new treatment modality is difficult. The setting of a randomised clinical trial with extensive follow-up and data safety monitoring seems to justify the decision. Should the prosthesis used in either group become dysfunctional, most patients will be candidates for either re-TAVI (valve-in-valve procedure) or surgical prosthesis replacement [21,33,34].

Patient eligibility for AV intervention and trial enrolment is evaluated consecutively at a multidisciplinary conference. In an attempt to explore the effect of TAVI in AV stenosis most specifically, patients with significant coronary artery disease requiring revascularisation at the time of referral are excluded. Study results will consequently not be readily applicable for a large group of AV stenosis patients requiring concomitant revascularisation. On the contrary, the study cohort should reflect patients seen in clinical practice, and therefore patients with previous, but not recent, myocardial or cerebral infarction are included. Patients with myocardial infarction and/or percutaneous coronary intervention with a drug-eluted stent within the previous year are excluded, however, since discontinuation of dual-antiplatelet therapy prior to surgery could expose these patients to a thromboembolic risk. Patients with atrial fibrillation are also included. To avoid redo-valve procedures due to expected prosthesis degeneration and mechanical valve prostheses, we chose the 70-year-old minimum age criterion.

Patients are excluded if they have undergone previous open cardiac surgery, since SAVR with re-sternotomy and mediastinal dissection after pericardiotomy represents a different and higher risk procedure than first-time SAVR.

Similar postprocedure antithrombotic therapy in both treatment groups was chosen to avoid the confounding effect of different medical antithromboembolic protection in the two intervention groups. This strategy was not in accordance with the guidelines at the time of designing the trial, which recommended 3 months of oral anticoagulation after SAVR [4]. No evidence existed for the optimal antithrombotic treatment after TAVI [35]. This situation has not changed substantially [5].

Only one TAVI system is used in the trial. Most centres use only one system as the technology is still new and requires a substantial amount of experience to be performed safely. However, as experience with more devices grows, a more complete picture of TAVI and its effectiveness should be evaluated in randomised trials comparing surgery with TAVI using all available delivery systems and prostheses.

We have tried to minimise bias in different ways. Selection bias is minimised by consecutive enrolment and central randomisation ensuring random allocation and allocation concealment [36,37]. The trial is not blinded owing to the inherently different nature of the two treatment modalities. Unblinded trials are at risk of introducing performance, collateral intervention, attrition, and assessment bias [36,37]. No investigator performs both interventions, but obvious differences in experience with a new procedure (TAVI) and a well-established procedure (SAVR) will introduce performance bias. Investigators performing TAVI have all done more than 75 procedures before this trial began. All cardiac surgeon investigators have extensive SAVR experience. The blinding of the event adjudication process for the primary outcome will diminish assessment bias. Publication of the trial protocol prior to analysing and reporting the data will prevent outcome reporting bias, and the multicentre design will improve external validity. The trial budget will unfortunately only accommodate 10% data monitoring, which obviously presents a limitation. Members of the independent DMSC and event adjudication committee have not been involved in any aspects of the trial design or conduct.

Other trials comparing new endovascular treatments against surgery have demonstrated that the overall benefit of a new procedure requires weighing of the relative risks and benefits in specific patient populations. There must be a reasonable balance between safety, effectiveness, and device durability. Safety may be more important than suboptimal effectiveness and durability for some patients. This concept demands patient assessment by an interventionalist and a cardiac surgeon before guidance can be given to the patient. Trials have thus suggested that surgery could perhaps be deferred as first-line treatment for certain high-operative-risk patients in myocardial revascularisation and mitral valve repair. This suggestion is also in accordance with patient preferences and wishes toward less invasive treatment options.

In conclusion, the NOTION trial is designed to compare the safety and effectiveness of TAVI versus conventional SAVR in the treatment of patients 70 years of age or older with stand-alone severe degenerative AV stenosis. TAVI may reduce postoperative morbidity and mortality without compromising a favourable functional outcome. This could expand the patient population referred for AV intervention and change the type of intervention used.

## Trial status

Since screening began in December 2009 and up to December 2012, 269 patients have been enrolled. Enrolment is expected to be complete at the end of January 2013.

## Abbreviations

AV: aortic valve; DMSC: data monitoring and safety committee; ECG: electrocardiogram; EuroSCORE: European System for Cardiac Operative Risk Evaluation; NOTION: Nordic Aortic Valve Intervention; SAVR: surgical aortic valve replacement; STS: Society of Thoracic Surgery Predicted Risk of Mortality; TAVI: transcatheter aortic valve implantation.

## Competing interests

Principal investigator LS serves as a physician proctor for Medtronic Inc. This role implies supervising other centres in implementing the TAVI device and implantation system. LS has received consulting and lecture fees, grant support, and reimbursement for travel expenses from Medtronic Inc. All remaining authors declare that they have no competing interests.

## Authors’ contributions

Authors HGT, LS, and DAS conceived and designed the study, acquired data, and drafted the manuscript. All other authors acquired data and/or made substantial contributions and revisions to the manuscript. All authors read and approved the final manuscript.
